# Oncogenic roles of GPR176 in breast cancer: a potential marker of aggressiveness and a potential target of gene therapy

**DOI:** 10.1007/s12094-023-03174-w

**Published:** 2023-04-20

**Authors:** Wen-jing Yun, Hang Xue, Ning Yang, Li-jun Xiao, Hong-zhi Sun, Hua-chuan Zheng

**Affiliations:** 1 Department of Oncology and Central Laboratory, The Affiliated Hospital of Chengde Medical University, Chengde, 067000 China; 2 Department of Immunology, Basic Medical College of Chengde Medical University, Chengde, 067000 China; 3 Cancer Center, The First Affiliated Hospital of Jinzhou Medical University, Jinzhou, 121001 China

**Keywords:** Breast cancer, GPR176, Aggressiveness, Prognosis, Target therapy

## Abstract

**Background:**

Belonging to the G-protein coupled receptor 1 family, G protein-coupled receptor 176 (GPR176) is associated with the Gz/Gx G-protein subclass and is capable of decreasing cAMP production.

**Methods:**

GPR176 expression was detected by qRT-PCR, bioinformatics analysis, Western blot and immunohistochemistry, and compared with clinicopathological characteristics of breast cancer. GPR176-related genes and pathways were subjected to bioinformatic analysis. We also explored the effects of GPR176 on the phenotypes of breast cancer cells.

**Results:**

Lower expression of GPR176 mRNA was seen in breast cancer than in normal tissues, but the opposite pattern was found for its protein (p < 0.05). GPR176 mRNA was associated with female sex, low T staging, non-Her-2^+^ subtypes, non-mutant p53 status in breast cancer (p < 0.05). GPR176 methylation was negatively correlated with its mRNA level and T staging in breast cancer, and was higher in breast cancer than normal tissues (p < 0.05). GPR176 protein expression was positively correlated with older age, small tumor size, and non-luminal-B subtype of breast cancers (p < 0.05). The differential genes of GPR176 were involved in receptor-ligand interaction, RNA maturation, and so forth (p < 0.05). GPR176-related genes were categorized into cell mobility, membrane structure, and so on (p < 0.05). GPR176 knockdown weakened the proliferation, glucose catabolism, anti-apoptosis, anti-pyroptosis, migration, invasion, and epithelial-mesenchymal transition of breast cancer cells.

**Conclusion:**

These results indicate that GPR176 might be involved in the tumorigenesis and subsequent progression of breast cancer by deteriorating aggressive phenotypes. It might be utilized as a potential biomarker to indicate the aggressive behaviors and poor prognosis of breast cancer and a potential target of genetic therapy.

## Introduction

The incidence and mortality of breast cancer (BC) have increased significantly over the past three decades, making it the most common form of cancer among women globally. BC risk factors may include a median age at menarche and first pregnancy, menopause at an older age, elevated estradiol levels, stress, Klinefelter syndrome, obesity, coffee intake, radiation exposure, gynecomastia, a family history of breast cancer, as well as BRCA2 and BRCA1 mutations. mRNA gene expression levels can be used to differentiate BC into four distinct molecular subtypes, namely Luminal A, Luminal B, HER2-enriched, and basal-like. Management of breast cancer is intricate and necessitates the use of multiple approaches including surgery, radiation therapy, chemotherapy, hormonal therapy, or biological treatments, which can be administered in various sequences. We endeavored to expand our comprehension of GPR176’s role in BC by exploring its effect on tissue, cell and molecular dynamics. This was done in order to enhance diagnosis, therapy and prevention through the detection of unique biomarkers and treatment objectives [1, 2].

G-protein-coupled receptor (GPCRs) are an essential component of many physiological processes, having seven membrane-spanning helices and being divided into five major categories: glutamate, rhodopsin, adhesion, frizzled, and secretin [3]. GPCRs can bind with natural ligands and then primarily couple to Gα, which can regulate critical effectors and generate some secondary messengers, and then trigger downstream signal pathways [4]. Structurally, GPCRs possess active and inactive conformations, and they fluctuate between the two spontaneously, without the need for agonists, at a baseline activity. Upon interaction with a ligand (from cyclic AMP to peptides and large proteins), GPCRs undergo a conformational alteration that activates heterotrimeric G proteins (guanine nucleotide-binding proteins), which transmits the extracellular to intracellular signals. Traditionally, GPCR kinases and β-arrestins act in unison to manage desensitization and trafficking of GPCR via ubiquitination, phosphorylation, and β-arrestins [5] thus regulating GPCR signaling. As reviewed, Fredriksson et al. [3] identified over 800 GPCRs sequences and 342 unique functional sequences, which make them good candidate drug targets for treating diseases [6].

G protein-coupled receptor 176 (GPR176), also called HB-954 and Gm1012, belongs to the G-protein coupled receptor 1 family. GPR176 gene is located to human chromosome 15q14-q15.1 and encodes 515-aa protein [7]. It has been revealed by Wang et al. [8] that endogenous GPR176 has four asparagine residues in its N-terminal region that are N-glycosylated. Additionally, GPR176 has a constitutive activity that is not dependent on an agonist, resulting in a decrease in cAMP synthesis. Doi et al. [9] demonstrated that GPR176 suppressed the cAMP signal pathway by coupling with the unique G-protein subclass Gz, which was mainly expressed in brains. Research [10] has shown that Gpr176 is the regulator of the circadian clock in the suprachiasmatic nucleus. GPR176 undergoes asparagine (N)-linked glycosylation, essential for the proper cell-surface expression. N-glycosylation of GPR176 and its downstream G-protein signal regulated human chronotypes [11].

Significantly, changes in the conserved N-glycosylation sites of human GPR176 (rs1473415441 and rs761894953) caused by missense variations had an effect on N-glycosylation, which in turn, reduced the protein expression and cAMP-repressive activity in the cells. Kakarala et al. [12] found that GPR176 might interact with free fatty acids as a ligand. Schultz et al. [13] demonstrated that the treatment of anacardic acid up-regulated GPR176 expression in breast cancer cells (MCF-7 and MDA-MB-231) according to RNA-seq. Forest et al. [14] revealed that GPR176 expression was more prominent in tumors of diffuse malignant epithelioid mesothelioma of a high grade. To find out the potential biomarkers of aggressiveness and prognosis and the potential target of gene therapy, we for the first time attempted to clarify the clinical, pathological and prognostic implications of GPR176 in BC, as well as its molecular mechanisms.

## Materials and methods

### Cell culture and transfection

Breast cancer (BT474, MCF-7, MDA-MB-231, and SK-BR-3) and normal (MCF-10A) cell lines were purchased from the Cell Bank of the Chinese Academy of Sciences, Shanghai, China. They were cultured in RPMI 1640 medium supplemented with 10% FBS at 5% CO_2_ and 37 °C. SK-BR-3 cells were transfected with sh-GPR176 at 24 h after seeding on dishes, and selected by G418.

### Proliferation assay

Following the seeding of SK-BR-3 cells into 96-well plates at a rate of 5 × 10^3^ cells per well, and allowing them to adhere overnight, the CCK-8 solution (10 μL/well) was introduced into each well of the plate at 0 h, 24 h, 48 h, and 72 h intervals. Then, the plate was incubated for 1 h in an incubator, after which absorbance at 450 nm was measured to assess the viable cell quantity.

### Metabolism assay

Cells were seeded at a density of 2.0 × 10^4^ cells/well into XF-24 Extracellular Flux Analyzers (Seahorse Bioscience, North Billerica, MA, USA). Subsequently, extracellular acidification and oxygen consumption rates were measured in XF media, which contained DMEM, 1 mM sodium pyruvate, either 10 mM or 15 mM glucose, and 2 mM l-glutamine, in the presence of mitochondrial inhibitors (1 mM antimycin A + 1 mM oligomycin and/or 100 nM rotenone).

### Apoptosis assay by flow cytometry

Flow cytometry with 7-AAD and APC-labeled Annexin V (BD Pharmingen, USA) was executed in line with the protocol to recognize phosphatidylserine externalization, a sign of early apoptosis.

### Wound healing assay

SK-BR-3 cells were cultured in six-well plates at a density of 2.0 × 10^5^ cells/well until confluence. To create a scratch, a yellow pipette tip was used to scrape the cell monolayer, followed by thrice washing with PBS and incubation in FBS-free medium. After 24 h and 48 h, the scratched area was measured using Image J software and photographs were taken.

### Cell migration and invasion assays

Five thousand cells were seeded in the upper chamber of a BD Bioscience migration assay, which was incubated with serum-free RPMI 1640. A chemoattractant of 10% FBS was added to the lower chamber. After 24 h, cells that had not passed through the membrane were removed with a cotton swab, washed with PBS, fixed in methanol and stained with Giemsa dye. For the determination of invasion experiments, the same procedure was followed, except Matrigel-coated inserts (BD Bioscience) were used.

### Patients

The paraffin-embedded blocks of breast cancer (n = 225) and normal tissues (n = 58) were collected from surgically resected specimens at The First Affiliated Hospital of Jinzhou Medical University (China). Fresh specimens (including adjacent normal tissues, n = 74) were collected from The Affiliated Hospital of Chengde Medical University and stored at − 80 °C from 2020 to 2021. Those samples were used for protein and RNA extraction. Before surgery, no patients had undergone chemotherapy, radiotherapy, or adjuvant treatment. Prior to the start of the clinical study, all patients provided written informed consent. The clinical research was approved by the ethics committee of the Affiliated Hospital of Chengde Medical University.

### Tissue microarray (TMA)

Pathological specimens were prepared by fixing them in 4% paraformaldehyde, followed by dehydration with alcohol, dealcoholization with xylene, and embedding in paraffin. Subsequently, 4 μm paraffin sections were cut and hematoxylin-and-eosin staining was employed for histological examination. Representative areas of adjacent normal tissues and solid tumors were identified under a microscope and corresponding tissue cores were punched out from paraffin blocks and transferred to pathological blocks, which were cut into 4-μm-thick sections.

### qRT-PCR

Total RNA of fresh specimens was isolated using RNAeasy Mini Kit (74104; QIAGEN, Germany), quantified, and then cDNA was reverse-transcribed using M-MLV and random primers (Takara, Japan). In line with sequences from GenBank, real-time PCR primers were designed by primer-BLAST in NCBI as follows: GAPDH primers: forward: 5′-CAATGACCCCTTCATTGACC-3′, reverse: 5′-TGGAAGATGGTGATGGGATT-3′; GPR176 primers: forward: 5′-TCCCTGCTATTGCTTTGGAC-3′, reverse: 5′-TACTGCAAACACAGGGACAC-3′.

### Western blotting

Fresh samples were lysed using RIPA buffer to extract total proteins, which were then separated by 10% SDS-PAGE and transferred to PVDF membranes in equal volumes. Nonspecific antigen sites were blocked with 5% skim milk for 1.5 h and then incubated with anti-GPR176 (1:1000, ab122605; Abcam, USA), rabbit anti-PTEN (1:2000; CST), rabbit anti- PI3K (1:1000; CST), rabbit anti-mTOR (1:1000; CST), mouse anti-Bcl-2 (1:500; Santa Cruz), mouse anti-Bax (1:500; Santa Cruz), rabbit anti-p-p38 (1:1000; Santa Cruz), rabbit anti-Caspase-1 (1:2000; Abcam), rabbit anti-E-cadherin (1:5000; Abcam), mouse anti-N-cadherin (1:2000; Abcam), rabbit anti-Zeb1 (1:1000; ABclonal), rabbit anti-Snail (1:1000; HuaBio), rabbit anti-Twist1 (1:1000; Wanleibio), mouse anti-ki-67 (1:1000; CST), mouse anti-β-actin (1:2000; Proteintech), mouse anti-p-mTOR (1:2000; Proteintech), rabbit anti-Stat3 (1:1000; Wanleibio), rabbit anti-p-Stat3 (1:1000; Wanleibio), mouse anti-PARP-1 (1:500; Santa Cruz), mouse anti-β-catenin (1:2000; Proteintech), rabbit anti-p-β-catenin (1:2000; CST), rabbit anti-Slug (1:5000; Abcam), rabbit anti-α-SMA (1:1000; Huaan), rabbit anti-MMP1 (1:1000; Wanleibio), rabbit anti-MMP9 (1:2000; Proteintech), rabbit anti-VEGF (1:500; Santa Cruz), or mouse anti-β-actin (1:2000; Proteintech) overnight at 4℃. After washing three times, the membranes were incubated with anti-rabbit or anti-mouse antibody with horseradish peroxidase (1:5000; CST) for 2 h. C300 (Azure Biosystems) was used to capture protein bands, and Western BrightTM ECL western blotting detection kit (Advansta) was employed for detection, Image J software (v1.8.0) providing the analysis.

### Immunohistochemistry (IHC)

The slides were subjected to three cycles of deparaffinization and rehydration, with antigen retrieval being conducted in a microwave oven for 20 min. Subsequently, to inhibit endogenous peroxidase activity, 3% hydrogen peroxide (H_2_O_2_) was applied, followed by 5% bovine serum albumin (BSA) to prevent non-specific binding sites for a duration of 30 min. Subsequently, slides were incubated with rabbit anti-GPR176 (1:80; Abcam) and mouse anti-p53 antibody (1:100; DAKO) for 1 h at room temperature. After being rinsed with PBS three times, the slides were exposed to polyclonal swine anti-rabbit or anti-mouse antibody with HRP (1:200; DAKO) at room temperature for a period of 2 h. Utilizing Diaminobenzidine (DAB), the specific binding sites were made visible. The slides, after being stained with hematoxylin, were dehydrated, cleared, and mounted, then viewed under a microscope (Nikon Corporation, Japan).

The cytoplasmic localization of GPR176 was evaluated by counting 100 randomly selected cells from five representative fields. The positivity rate was then determined by two researchers (YWJ and ZHC) as follows: 0 = 0%; 1 = 1–49%; 2 = 50–74%; and 3 ≥ 75%. The intensity levels were classified as follows: 1 = weak; 2 = medium; and 3 = strong. The immunohistochemical score was calculated as intensity × positive rate, with the scores defined as follows: −  = 0; +  = 1–2; ++  = 3–5; and +++  = 6–9.

### Bioinformatic analysis

The expression, methylation, related genes, and signal pathways of the GPR176 gene were analyzed with the xiantao platform (https://www.xiantao.love/), Timer (https://cistrome.shinyapps.io/timer/), UALCAN database (http://ualcan.path.uab.edu), and cBioPortal (https://www.cbioportal.org). The xiantao platform has efficiently optimized and encapsulated common statistical analysis and visualization functions in the R language. It has also incorporated front-end technology, which enables one-click visualization, thereby effectively addressing the medical big data analysis and processing requirements. The TIMER database, which utilizes high-throughput sequencing (RNA-Seq expression profile) data, is utilized to analyze the infiltration of immune cells in tumor tissues. It primarily focuses on the infiltration of six types of immune cells, including B cells, CD4 + T cells, CD8 + T cells, Neutrophils, Macrophages, and Dendritic cells. UALCAN is an interactive web resource that provides easy access to TCGA's publicly available cancer-omics data, allowing users to identify biomarkers or electronically validate potential genes of interest. It is a comprehensive tool that can help analyze cancer omics data. Additionally, cBioPortal integrates data from 126 tumor genome studies, including large tumor studies such as TCGA and ICGC, covering data from 28,000 samples, some of which also include clinical prognostic and phenotypic information. The prognostic significance of GPR176 was explored using Kaplan–Meier Plotter (http://kmplot.com/). Kaplan–Meier Plotter data derived from GEO, EGA, and TCGA databases, were able to assess the correlation between the expression of all genes and patient survival in more than 30,000 samples from 21 tumors to identify and validate survival-related biomarkers. Additionally, we identified the differentially expressed genes and genes related to GPR176 using Xiantao. The differentially expressed genes were used for the construction of a PPI network and selected as important hub genes. These genes were subjected to GO + KEGG and GSEA analysis for the construction of signal pathways.

### Statistical analysis

To assess the means, rank data, and rates, Mann–Whitney U, Spearman’s, and chi-squared tests were used. The log-rank statistic was used to compare the survival curves, resulting in the creation of Kaplan–Meier survival plots. Data processing and statistical analysis was conducted using SPSS 20.0 software, with P < 0.05 being established as the criterion for determining significance.

## Results

### Clinicopathological and prognostic significances of GPR176 mRNA expression in breast cancer

**Fig. 1:**
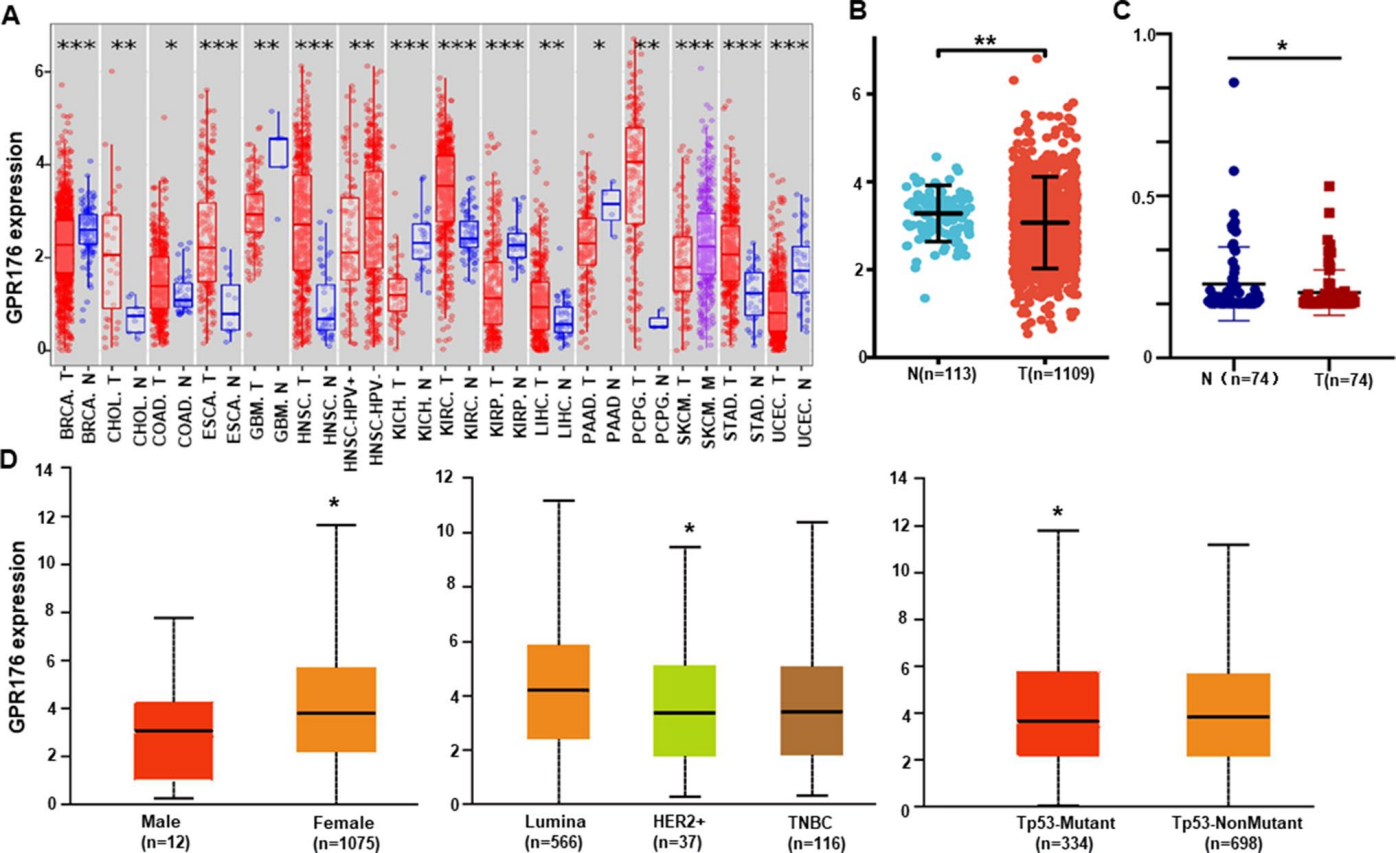
The clinicopathological significances of GPR176 mRNA expression in breast cancer. We evaluated the GPR176 mRNA expression in pan-cancer using Timer (**A**). GPR176 mRNA was analyzed in breast cancer using xiantao platform (**B**) and real-time PCR (**C**). It was also compared with clinicopathological features of breast cancer using UALCAN database (D). *N* normal tissue, *T* tumor, *M* metastasis, *BRCA* breast invasive carcinoma, *CHOL* cholangiocarcinoma, *COAD* colon adenocarcinoma, *ESCA* esophageal adenocarcinoma, *GBM* glioblastoma multiform, *HNSC* head and neck squamous cell carcinoma, *HPV* human papillomavirus, *KICH* kidney chromophobe, *KIRC* kidney renal clear cell carcinoma, *KIRP* kidney renal papillary cell carcinoma, *LIHC* liver hepatocellular carcinoma, *PAAD* pancreatic adenocarcinoma, *PCPG* pheochromocytoma and paraganglioma, *SKCM* skin cutaneous melanoma, *STAD* stomach adenocarcinoma, *UCEC* uterine corpus endometrial carcinoma, *TNBC* triple-negative breast cancer. ***p < 0.01; **p < 0.01; *p < 0.05

According to Timer, we found that GPR176 mRNA was more highly expressed in cholangiocarcinoma, colon adenocarcinoma, esophageal carcinoma, head and neck squamous cell carcinoma (HNSCC), kidney renal clear cell carcinoma, hepatocellular carcinoma, pheochromocytoma and paraganglioma, and gastric adenocarcinoma than in normal tissues (p < 0.05, Fig. 1A), while it was expressed at lower levels in invasive breast carcinoma, glioblastoma multiforme, kidney chromophobe, kidney renal papillary cell carcinoma, pancreatic adenocarcinoma, and uterine corpus endometrial carcinoma (p < 0.05, Fig. 1A). It was associated with a lack of HPV (human papillomavirus) infection in HNSCC, and metastasis of skin melanoma (p < 0.05, Fig. 1A). Meanwhile, its low expression in breast cancer was confirmed by the xiantao database (p < 0.05, Fig. 1B) and real-time RT-PCR (p < 0.05, Fig. 1C). According to the UALCAN database, GPR176 mRNA expression was higher in female than in male patients, and in Luminal and TNBC (triple-negative breast cancer) than in Her-2-positive cases, as well as in patients with nonmutant than mutant p53 (Fig. 1D, p < 0.05). As shown in Table 1, GPR176 mRNA expression was also negatively correlated to the T staging of breast cancer (p < 0.05).

**Table 1:** Relationship between GPR176 mRNA expression and clinicopathological characteristics of breast cancer

Characteristics	Variables	Low expression	High expression	*p*
Age (years), n (%)	≦ 60	286 (26.4%)	315 (29.1%)	0.093
	> 60	255 (23.5%)	227 (21%)	
T stage, n (%)	T1	119 (11%)	158 (14.6%)	**0.035**
	T2	322 (29.8%)	307 (28.4%)	
	T3	78 (7.2%)	61 (5.6%)	
	T4	20 (1.9%)	15 (1.4%)	
N stage, n (%)	N0	260 (24.4%)	254 (23.9%)	0.112
	N1	188 (17.7%)	170 (16%)	
	N2	46 (4.3%)	70 (6.6%)	
	N3	37 (3.5%)	39 (3.7%)	
M stage, n (%)	M0	441 (47.8%)	461 (50%)	0.753
	M1	11 (1.2%)	9 (1%)	
Pathologic stage, n (%)	Stage I	85 (8%)	96 (9.1%)	0.518
	Stage II	322 (30.4%)	297 (28%)	
	Stage III	115 (10.8%)	127 (12%)	
	Stage IV	9 (0.8%)	9 (0.8%)	
Histological type, n (%)	IDC	383 (39.2%)	389 (39.8%)	0.797
	ILC	99 (10.1%)	106 (10.8%)	
PR status, n (%)	Negative	174 (16.8%)	168 (16.2%)	0.903
	Indeterminate	2 (0.2%)	2 (0.2%)	
	Positive	341 (33%)	347 (33.6%)	
ER status, n (%)	Negative	117 (11.3%)	123 (11.9%)	0.448
	Indeterminate	2 (0.2%)	0 (0%)	
	Positive	399 (38.6%)	394 (38.1%)	
HER2 status, n (%)	Negative	262 (36%)	296 (40.7%)	0.314
	Indeterminate	5 (0.7%)	7 (1%)	
	Positive	84 (11.6%)	73 (10%)	
PAM50, n (%)	Normal	15 (1.4%)	25 (2.3%)	0.231
	Lum A	275 (25.4%)	287 (26.5%)	
	Lum B	109 (10.1%)	95 (8.8%)	
	HER2 +	47 (4.3%)	35 (3.2%)	
	Basal	95 (8.8%)	100 (9.2%)	

*IDC* invasive intraductal carcinoma, *ILC* invasive lobular carcinoma, *PAM50* prediction analysis of microarray 50, *ER* estrogen receptor, *PR* progesterone receptor, *P*-values in bold p <0.05

**Fig. 2:**
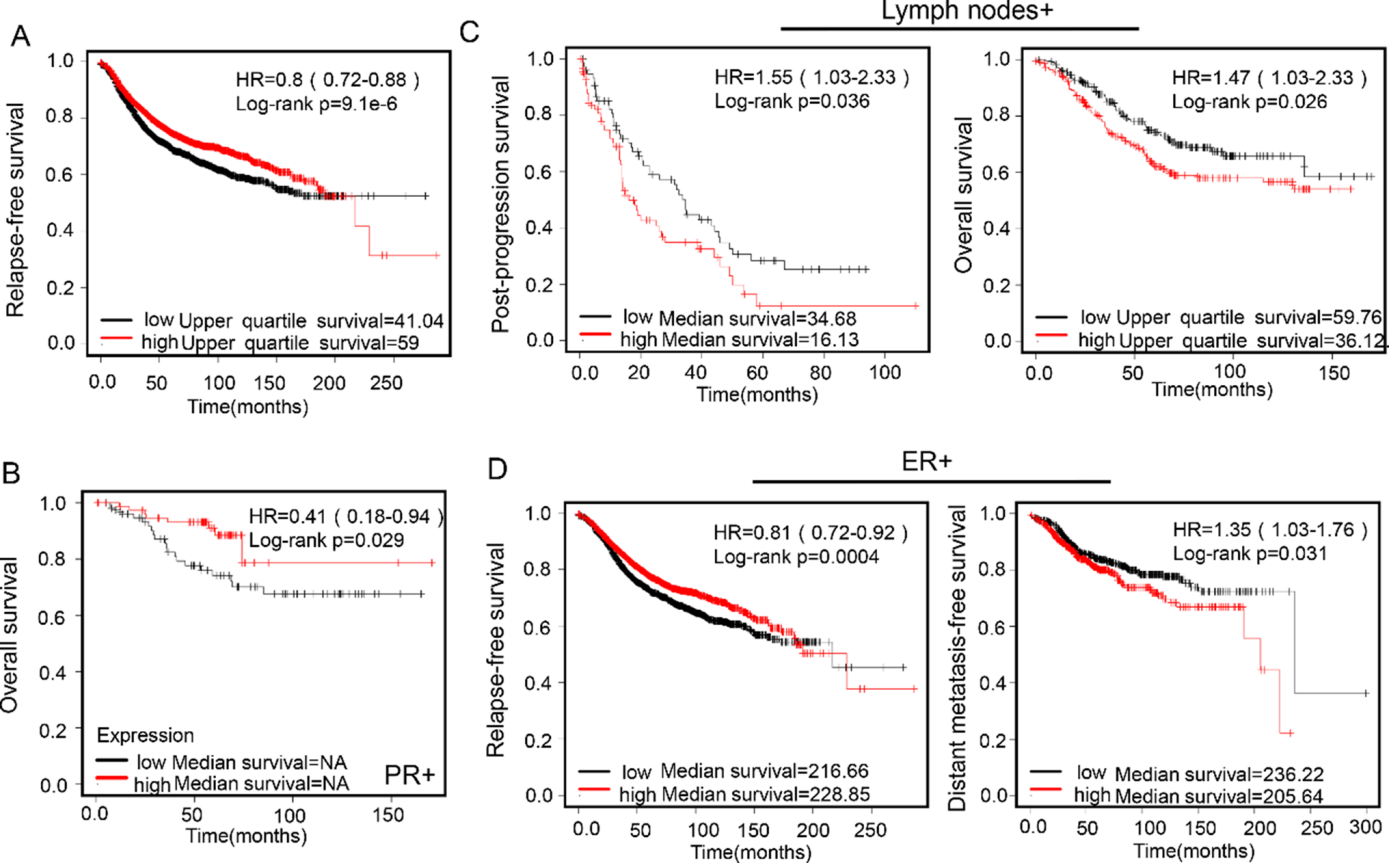
The prognostic significance of GPR176 mRNA expression in breast cancer by Kaplan–Meier plotter. *ER* estrogen receptor, *PR* progesterone receptor, *HR* hazard ratio

Kaplan–Meier Plotter showed that GPR176 mRNA expression was positively correlated with the relapse-free survival (RFS) of all cancer patients (Fig. 2A, p < 0.05) and the overall survival (OS) of PR (progesterone receptor)-positive cases (Fig. 2B, p < 0.05). There was a negative relationship between GPR176 mRNA expression and OS or post-progression survival of the cancer patients with lymph node involvement (Fig. 2C, p < 0.05). In the ER (estrogen receptor)-positive cases, GPR176 mRNA expression was positively associated with RFS, but negatively associated with distant-metastasis-free survival (Fig. 2D, p < 0.05).

**Fig. 3:**
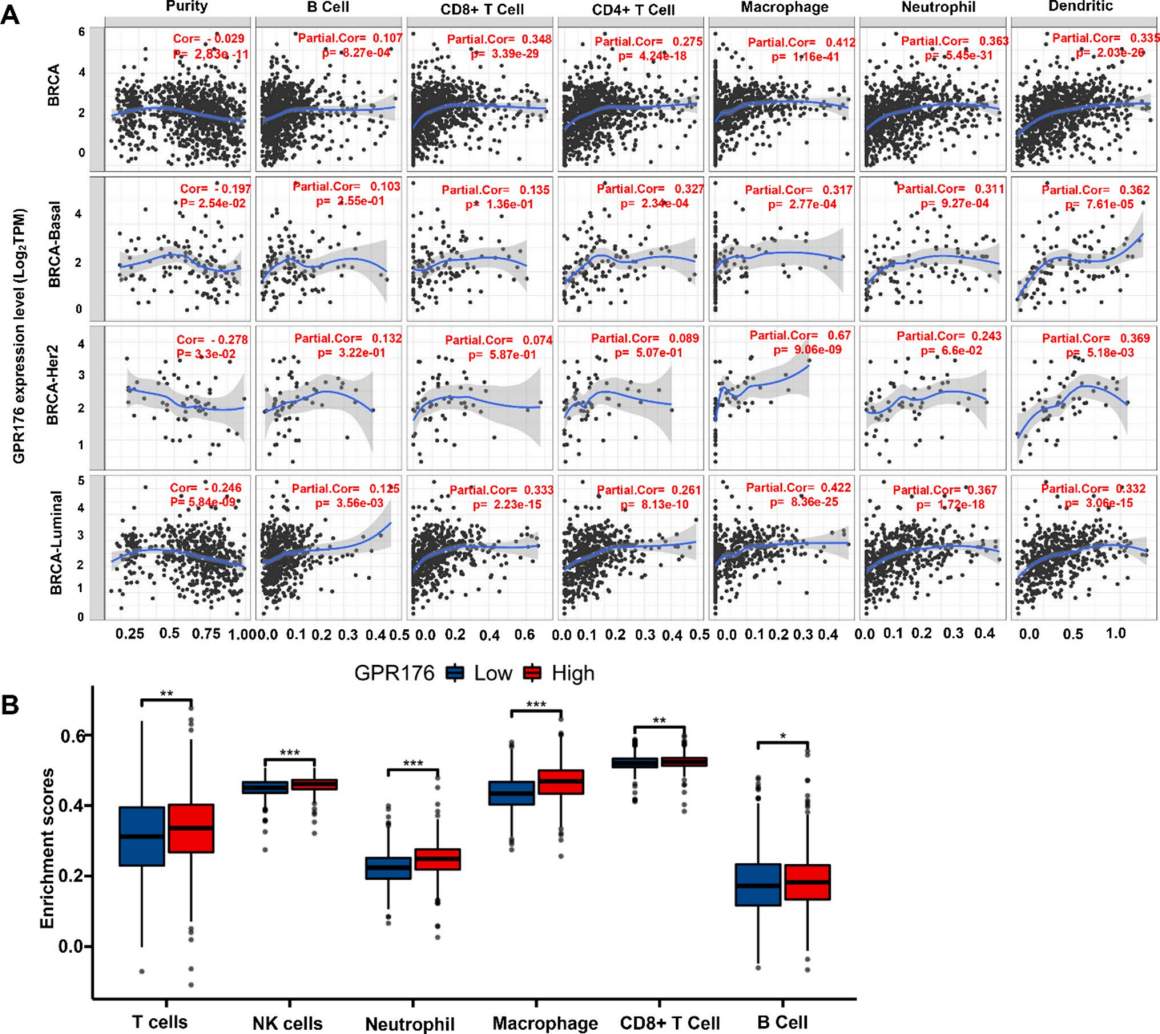
The relationship between GPR176 mRNA expression and immune infiltration in breast cancer. The correlation was studied between the infiltration of immune cells and GPR176 mRNA expression in different subtypes of breast cancer using Timer (**A**). The enrichment of immune cells was explored between low and high expression of GPR176 in breast cancer using xiantao (**B**). *BRCA* breast cancer

According to Timer, GPR176 mRNA expression was negatively related to purity in BRCA, BRCA-basal, BRCA-Her2, and BRCA-Luminal (Fig. 3A, p < 0.05). In BRCA and BRCA-luminal subtypes, it was positively associated with the infiltration of B cells, CD4^+^ T cells, CD8^+^ T cells, macrophages, neutrophils, and dentritic cells (Fig. 3A, p < 0.05). In the basal-like subtype, it was positively associated with CD4^+^Tcells, macrophages, neutrophils, and dendritic cells (Fig. 3A, p < 0.05). In the Her2^+^ subtype, it was positively associated with the infiltration of macrophages and dendritic cells (Fig. 3A, p < 0.05). The same trends were observed in the infiltration of T cells, NK cells, neutrophils, macrophages, CD8^+^ T cells, and B cells, according to xiantao (Fig. 3B, p < 0.05).

### Correlation of GPR176 promoter methylation with carcinogenesis and pathological behaviors of breast cancer

**Fig. 4:**
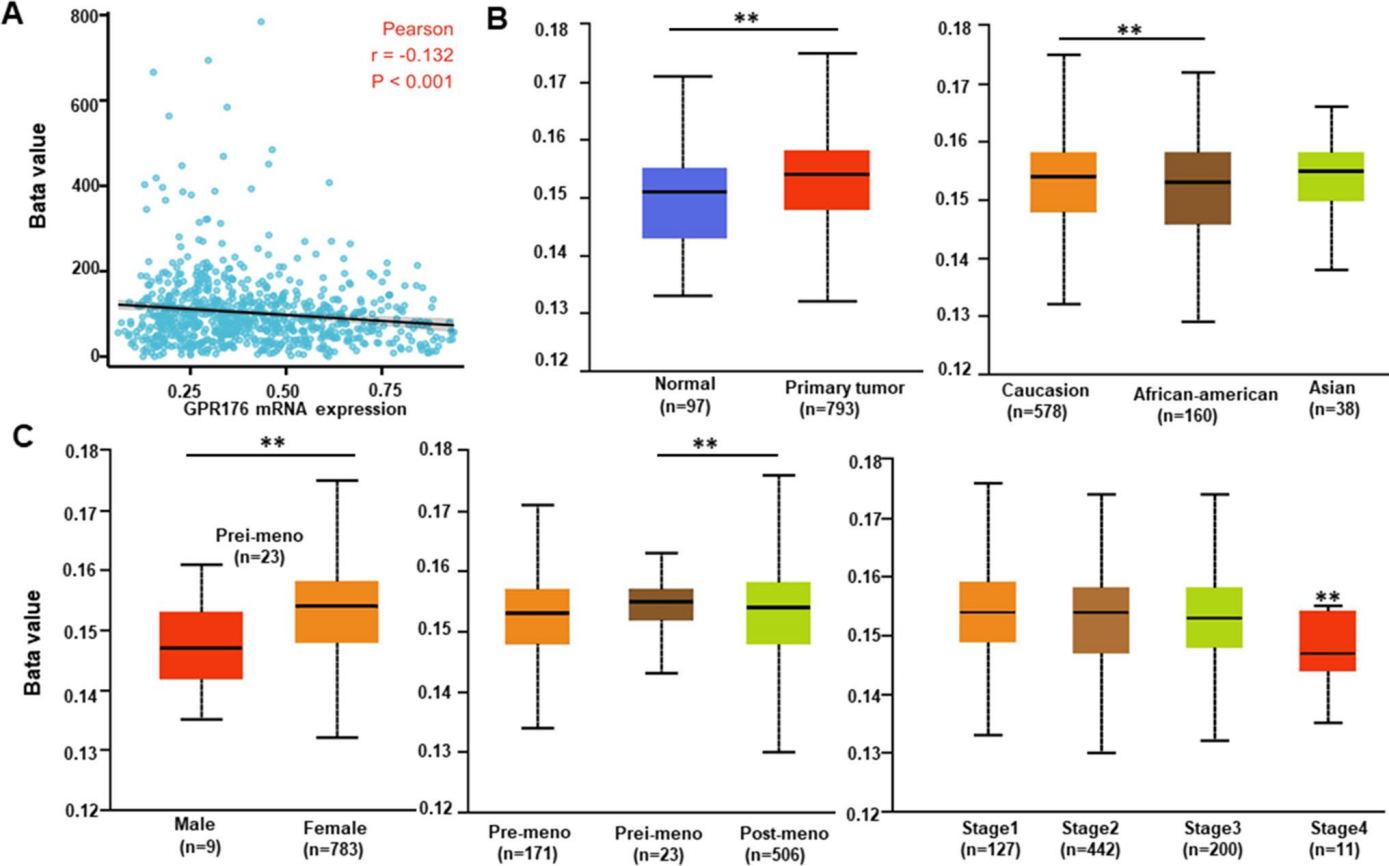
The clinicopathological significance of GPR176 methylation in breast cancer. The negative relationship between GPR176 mRNA expression and methylation was analyzed in breast cancer using cBioPortal database (**A**). Its methylation was higher in breast cancer than normal tissues (**B**). We also compared GPR176 methylation with clinicopathological characteristics of breast cancer (**C**)

There was a negative relationship between GPR176 mRNA expression and promoter methylation in breast cancer, according to the cBioPortal database (Fig. 4A, p < 0.05). GPR176 promoter methylation was higher in breast cancer than in normal tissues，in Caucasians than in African-Americans according to UALCAN (Fig. 4B, p < 0.05). In addition, as indicated in Fig. 4C, such methylation was higher in females than in males (p < 0.05), in peri-menopausal patients than in post-menopausal (p < 0.05), and in breast cancer patients with stages 1–3 than in those with stage 4, according to UALCAN (p < 0.05).

### Genes and signal pathways related to GPR176 in breast cancer

**Fig. 5:**
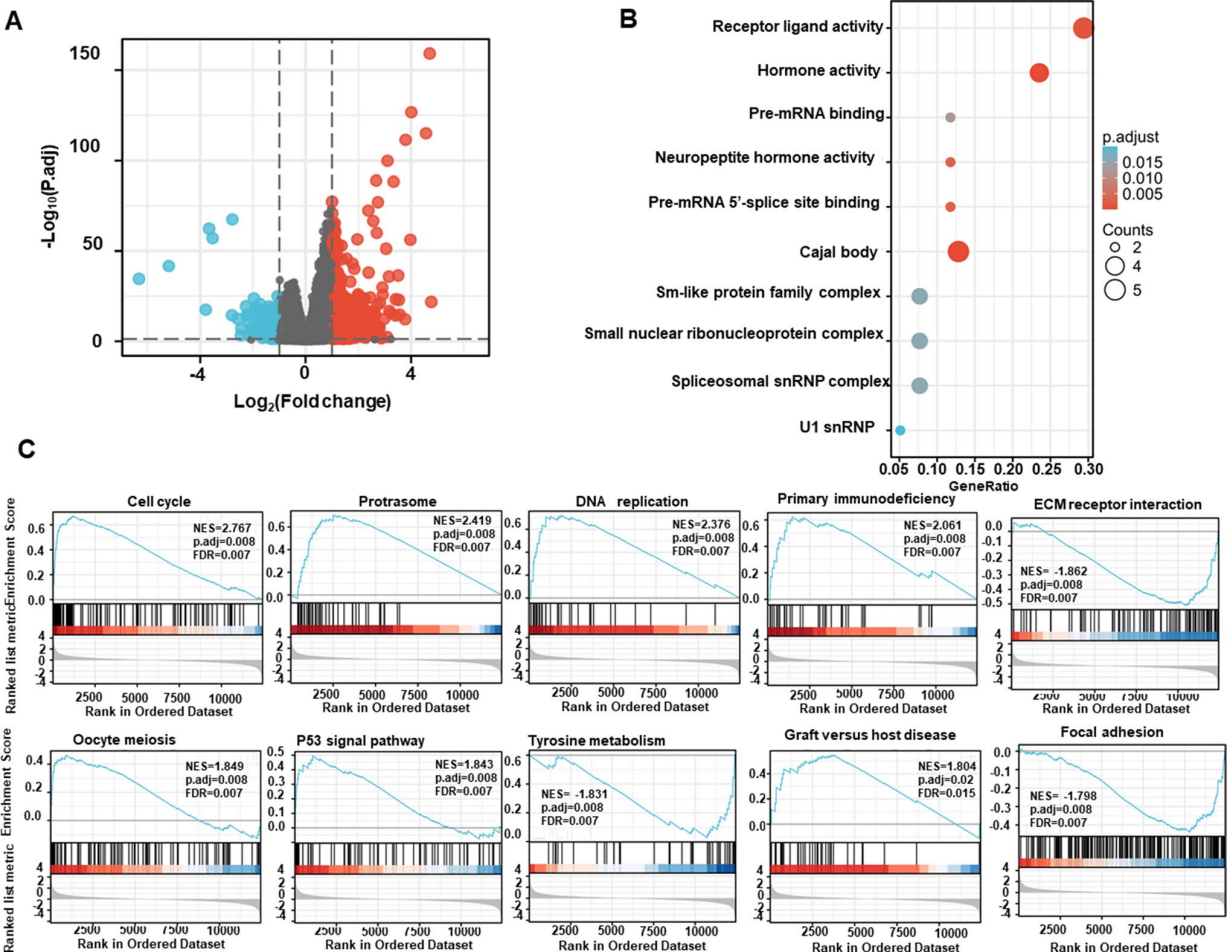
The differential genes and their signal pathways between low and high GPR176 expression in breast cancer. The hotmap of the differential genes was shown between low and high GPR176 expression in breast cancer (**A**). These genes were subjected to the signal pathway analysis using KEGG (**B**) and GSEA (**C**)

**Fig. 6:**
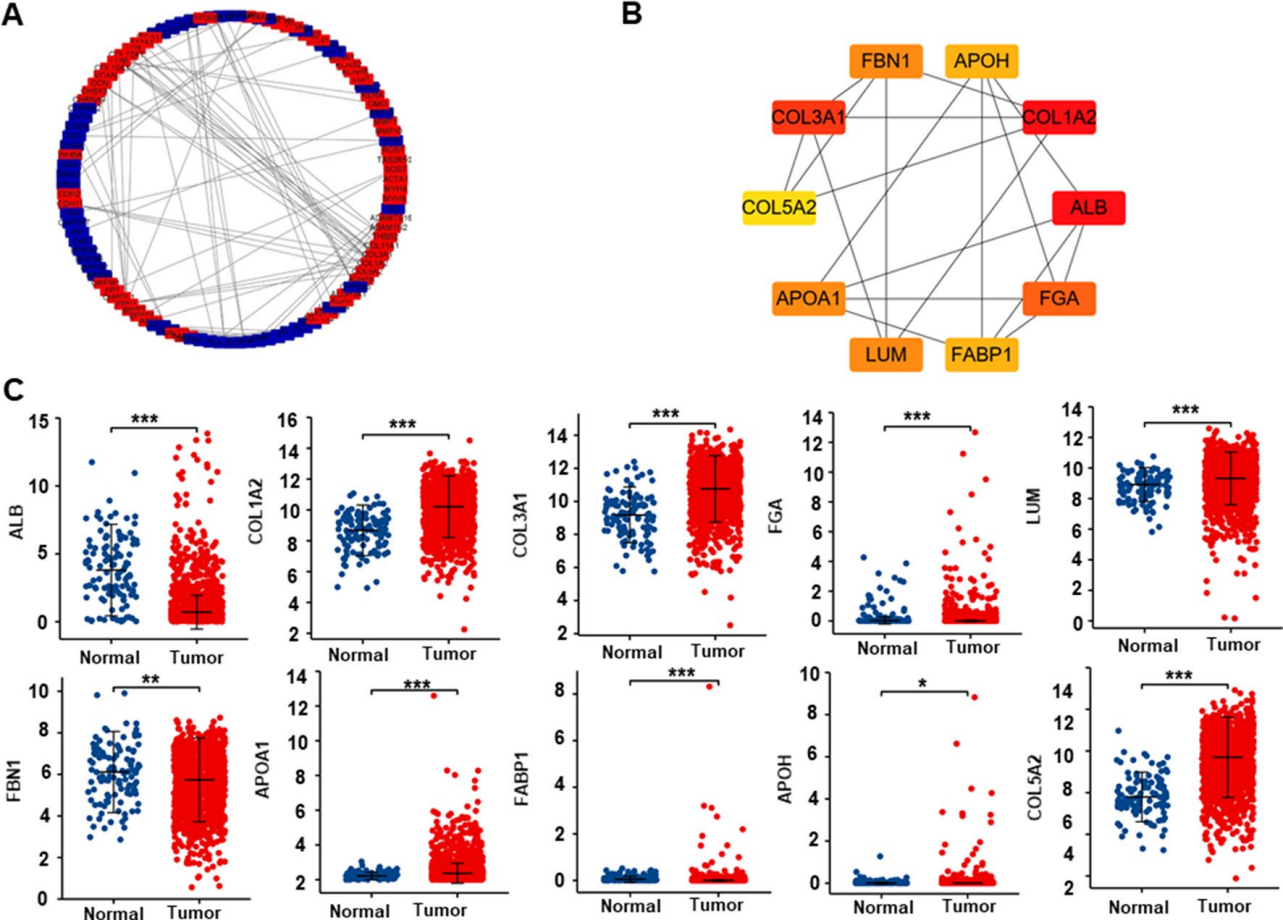
The hub genes of GPR176 in breast cancer. Both string and cytoscape were employed to screen the hub genes of GPR176 in breast cancer (**A**). The hotspot hub genes were selected (**B**) and compared between breast cancer and normal tissues (**C**)

In the xiantao platform, we found the genes differentially expressed between the groups with low and high expression of GPR176 mRNA in breast cancer used them to build a volcano plot, as shown in Fig. 5A. KEGG analysis showed that the top signal pathways included receptor-ligand interaction, RNA maturation, hormone, and sm-like protein family, among others (Fig. 5B, p < 0.05). GSEA showed that the top signal pathways comprised cell cycle, proteasome, DNA replication, ECM-receptor interaction, focal adhesion, and p53 signal pathway (Fig. 5C, p < 0.05). In addition, STRING was used to identify the PPI pairs (Fig. 6A) and cytoscape was used to find the top 10 nodes ranked by degree (Fig. 6B). According to the xiantao database, COL1A2, COL3A1, FGA, LUM, APOA1, APOH, and COL5A2 were more highly expressed in breast cancer than in normal tissues (Fig. 6C, p < 0.05), while the opposite pattern was seen for ALB and FBN1 (Fig. 6C, p < 0.05).

**Fig. 7:**
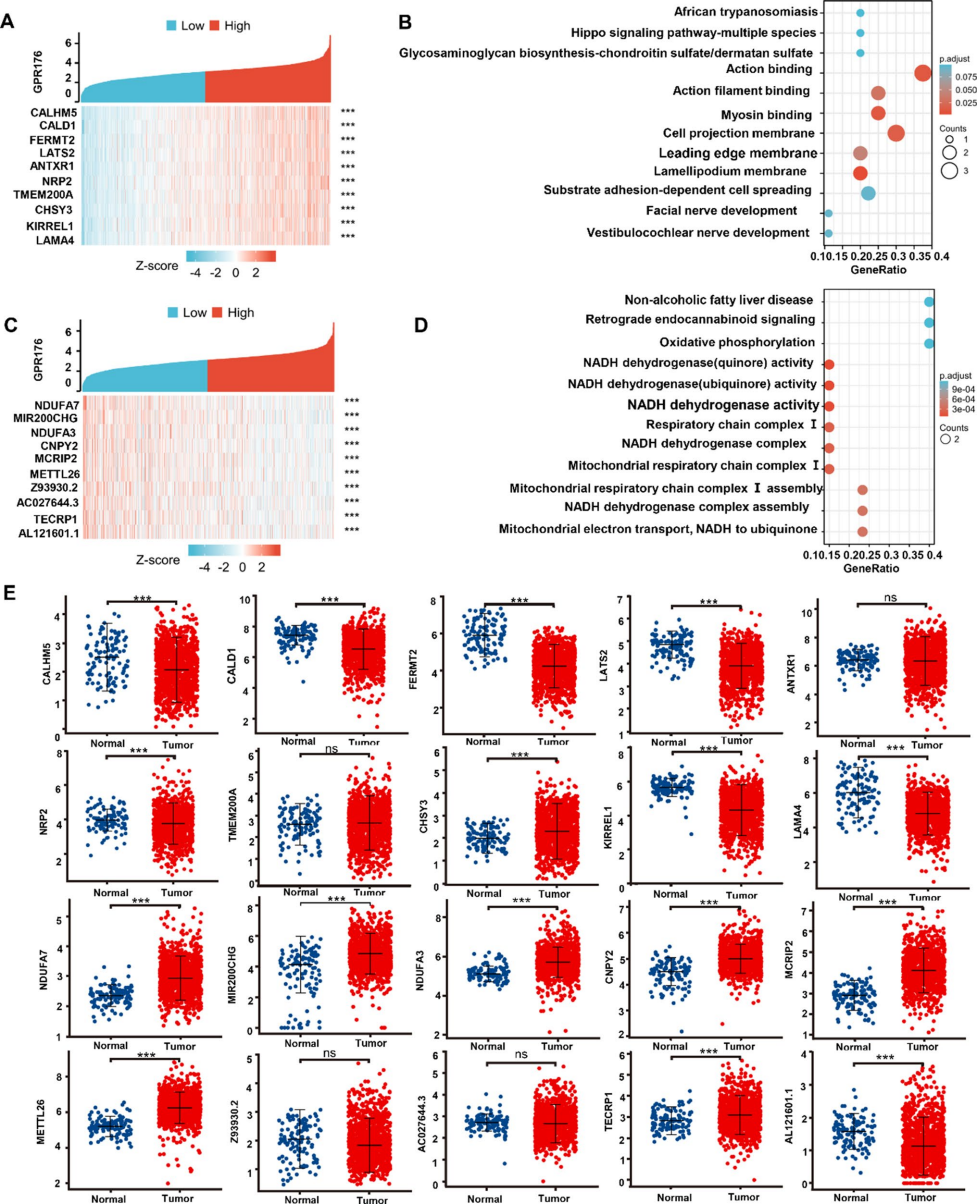
The GPR176-related genes and signal pathways in breast cancer. The positively-related genes of GPR176 were screened (**A**), and were classified into the signal pathway using xiantao database (**B**). The negatively-related genes of GPR176 were screened (**C**), and were classified into the signal pathway using xiantao database (**D**). The genes were compared between breast cancer and normal tissues using xiantao platform (**E**)

The genes whose expression was positively correlated with that of GPR176 in breast cancer according to the xiantao database are shown in Fig. 7A (p < 0.05). They are involved in cell mobility, membrane structure, nerve development, and Hippo signal pathway, among others (Fig. 7B). The genes whose expression was negatively correlated with that of GPR176 in breast cancer are shown in Fig. 7C (p < 0.05). They are involved in mitochondrial respiratory chain and non-alcoholic fatty diver disease, among others (Fig. 7D). The positively correlated genes (CALHM5, CALD1, FERMT2, LATS2, NRP2, KIRREL1, and LAMA4) were expressed less in breast cancer in than normal tissue (Fig. 7E, p < 0.05), while the converse was true for some of the negatively correlated genes (NDUFA7, MIR200CHG, NDUFA3, CNPY2, MCRIP2, METTL26, TECRP1) (Fig. 7E, p < 0.05).

### Clinicopathological significance of GPR176 protein expression in breast cancer

**Fig. 8:**
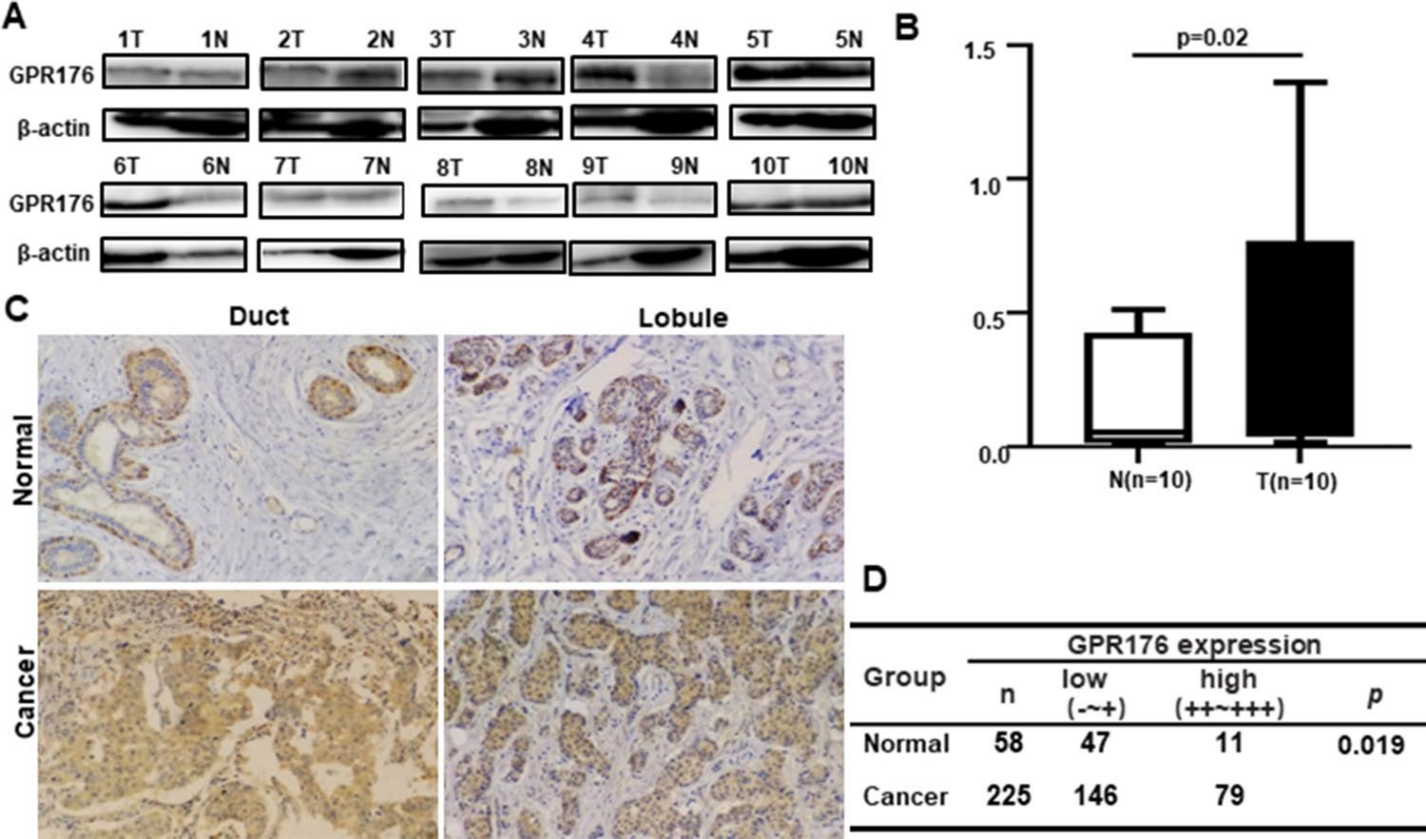
The clinicopathological significance of GPR176 protein expression in breast cancer. Western blot was used to detect GPR176 protein level in breast cancer (**A**). Densitometric analysis showed its higher expression in breast cancer than normal tissues (**B**, p < 0.05). Immunohistochemically, GPR176 protein was positively expressed in smooth muscle cell around breast ducts and lobules, and ductal and lobular adenocarcinoma, but weakly in ductal and lobular epithelium (**C**). Statistically, GPR176 protein expression was higher in breast cancer than normal tissues (**D**, p < 0.05). *N* normal, *T* tumor

According to a densitometric analysis of the western blot, GPR176 expression was higher in breast cancer than in matched normal tissues (Fig. 8A, B, p < 0.05). Immunohistochemically, GPR176 protein was strongly distributed in membrane and/or cytoplasm of smooth muscle cells around the ducts and lobules of the breast, and in ductal and lobular cancer, but weakly in ductal and lobular epithelial cells (Fig. 8C). GPR176 immunoreactivity was significantly lower in normal breast than in breast cancer (Fig. 8D, p < 0.05). In addition, GPR176 expression was associated with older age, smaller tumor size, and the non-luminal-B subtype of breast cancer (Table 2, p < 0.05).

**Table 2:** Relationship between GPR176 protein expression and clinicopathological characteristics of breast cancer

Clinicopathological characteristics	GPR176 expression			
	*n*	Low (− ~ +)	high(++ ~ +++)	*p*
Age(years)				**0.001**
≥ 50	53	24	29	
< 50	172	122	50	
Tumor size (cm)				**0.015**
< 2	55	27	28	
2–5	126	90	36	
> 5	14	9	5	
N stage				0.300
N0	51	37	14	
N1	23	20	3	
N2	11	10	1	
N3	2	2	0	
Pathological typing				0.405
DCIS	26	14	12	
IDC	154	102	52	
ILC	5	2	3	
Other types	22	15	7	
Histological grades				0.446
I–II	142	94	48	
II–III	16	12	4	
Molecular subtyping				**0.029**
Lum A	22	11	11	
Lum B	55	44	11	
HER2 +	9	5	4	
Basal-like	35	20	15	
P53 expression				
−	37	16	21	0.141
+	74	44	30	
++	5	4	1	

*DCIS* ductal carcinoma in situ, *IDC* invasive ductal carcinoma, *ILC* invasive lobular carcinoma, *P*-values in bold p<0.05

### Effects of GPR176 expression on the aggressive phenotypes of breast cancer cells

**Fig. 9:**
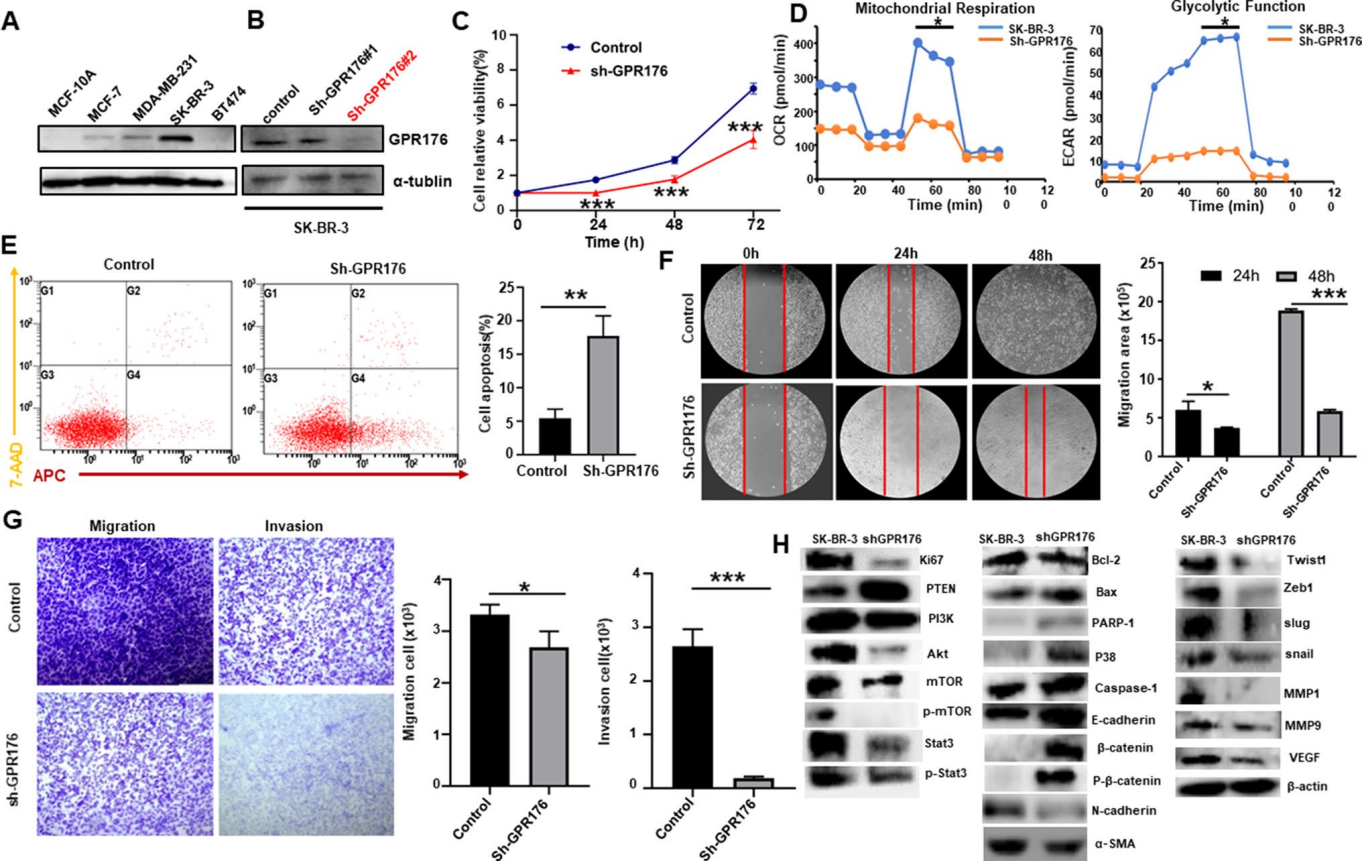
The effects of GPR176 expression on the phenotypes of breast cancer cells. GPR176 expression was screened in breast cancer cells by western blot (**A**). After transfection of sh-GPR176, GPR176 expression became weak in SK-BR-3 cells by Western blot (**B**). The transfectants (sh-GPR176#2) showed a slow proliferation, glycolysis and mitochondrial oxidation, a high apoptosis, lower migration and invasion in comparison with the control, evidenced by CCK-8 (**C**), seahorse energy examination (**D**), Annexin-V-APC/7-AAD staining (**E**), wound healing (**F**), and transwell assays (**G**). The phenotype-related proteins were screened by Western blot (**H**). Note: *, compared with the control, p < 0.05

GPR176 protein was strongly expressed in SK-BR-3 cells, but weakly in MCF-7 and MDA-MB-231 cells, and not expressed in MCF-10A and BT474 (Fig. 9A). Sh-GPR176 #1 and #2 silenced GPR176 expression in SK-BR-3 cells as determined by western blot and #2 was used in the subsequent experiments (Fig. 9B). GPR176 knockdown suppressed the proliferation, as evidenced by CCK-8 (Fig. 9C, p < 0.05), and glycolysis and mitochondrial oxidation by XF-24 extracellular flux analyzers (Fig. 9D, p < 0.05). Higher levels of apoptosis, and lower migration and invasion were found in GPR176 transfectants than in parental cells, according to Annexin-V staining (Fig. 9E, p < 0.05), wound healing (Fig. 9F, p < 0.05), and transwell chamber assays (Figs. 9G, p < 0.05). GPR176 knockdown increased the expression of PTEN, Bax, PARP-1, p38, Caspase-1, E-cadherin, β-catenin, and p-β-catenin, but decreased that of ki-67, PI3K, Akt, mTOR, p-mTOR, stat3, p-stat3, Bcl-2, N-cadherin, α-SMA, Twist1, Zeb1, slug, snail, MMP1, MMP9, and VEGF in SK-BR-3 cells (Fig. 9H).

## Discussion

Ligand-bound GPRs undergo conformational changes and thus act as guanosine exchange factors (GEFs). The α subunit of G-protein is separated from the β and γ subunits by exchanging GDP on the G protein with GTP. This process causes the G protein (specifically, its α subunit, which binds to GTP) to become active and involved in the next step of signaling. The specific pathway depends on the type of α subunit (GαS, GαI/O, GαQ/11, and Gα12/13), and the two main pathways involve cAMP or phosphatidylinositol. Nearly 1000 G-protein-coupled receptor genes have been predicted based on the sequence of the human genome. These G-protein-coupled receptors can be classified into six types, namely, Classes A (rhodopsin-like receptors), B (secretin receptor family), C (metabolic glutamate receptors), D (fungal mating pheromone receptors), E (cyclic adenylate receptors), and F (frizzled/smoothened family) [15–17].

Here, we found that GPR176 was downregulated in breast cancer by both real-time PCR and bioinformatics analysis, but upregulated by both western blot and immunohistochemistry, indicating that aberrant GPR176 expression plays an important role in breast carcinogenesis. This discrepancy might be due to the rapid translation of GPR176 mRNA and high stability of GPR176 protein in breast cancer. In breast cancer cells, the expression of GPR176 mRNA was noted to be inversely related to purity, but positively linked to the presence of immune cell infiltration. This observation points towards the potential contribution of GPR176 in immune surveillance, as well as the response of breast cancers to chemotherapy. Further research will be conducted to delve deeper into this matter. Moreover, GPR176 mRNA hypoexpression was closely linked to promoter hypermethylation in breast cancer, indicating that GPR176 methylation is responsible for its downregulated mRNA expression. These findings indicate that the aberrant expression and promoter methylation of GPR176 play important roles in breast carcinogenesis.

Additionally, we found that GPR176 expression was negatively associated with male sex, T staging, Her-2 positivity, and mutant p53 status of breast cancer, in line with the positive relationship between GPR176 methylation and pathological stage, and immunohistochemical findings. These results suggest that aberrant GPR176 expression and methylation might be useful as indicators of the clinicopathological behaviors of breast cancer. Additionally, upregulated GPR176 protein expression in breast carcinogenesis was downregulated with the progression of breast cancer, suggesting that GPR176 protein might be involved in breast carcinogenesis as an early event. During the progression of breast cancer, GPR176 expression might be regulated by other factors as a negative feedback regulation. Here, GPR176 silencing was found to suppress the proliferation, anti-apoptosis, migration and invasion of breast cancer cells. Taken together, we speculated that GPR176 might promote breast carcinogenesis by deteriorating the aggressive phenotypes of breast cancer cells.

GPR176 mRNA expression was demonstrated to positively correlate with the RFS of all or ER-positive cancer patients, and the OS of PR-positive cases, in line with the negative correlation between GPR176 expression and aggressive behaviors. Surprisingly, a negative relationship between GPR176 mRNA expression and OS, PPS, or DMFS was seen in the cancer patients with lymph node involvement or ER positivity, which might be linked to the post-surgery treatment, including endocrine and radiological therapies. Taking the obtained findings together, GPR176 might act as a biological marker to indicate the development of breast cancer.

Our bioinformatics analysis showed that the GPR176-related signal pathways included receptor-ligand interaction, RNA maturation, proteasome, DNA replication, ECM-receptor interaction, focal adhesion, cell mobility, membrane structure, Hippo signal pathway, mitochondrial respiratory chain, and non-alcoholic fatty diver disease. Human coronary artery smooth myocytes were treated with minimally oxidized LDL with GPR176 downregulated, in agreement with our finding [18]. In addition, we found that GPR176 knockdown might inhibit migration, invasion, and glycolysis and mitochondrial oxidation of glucose in breast cancer cells, indicating that GPR176 might exacerbate the aggressive phenotypes of breast cancer cells, including invasion and glucose consumption.

The PTEN/PI3K/Akt/mTOR pathway is one of the most frequently overactivated intracellular pathways and involved in proliferation and anti-apoptosis in various cancers [19]. In apoptosis, Bcl-2 can interact with Bax on the mitochondrial membrane to suppress Bax-mediated opening of the mitochondrial voltage-dependent anion channel for apoptosis [20]. In breast cancer cells, GPR176 silencing was shown to ameliorate proliferation and induce apoptosis by either inactivating PTEN/PI3K/Akt/mTOR or decreasing Bcl-2/Bax. Pyroptosis is a recently discovered form of inflammatory programmed necrosis characterized by caspase-1-mediated cell death [21]. Zeb1, Slug, Snail, and Twist1 have been found to promote epithelial–mesenchymal transition (EMT) with E-cadherin overexpression, and N-cadherin and α-SMA underexpression [22]. Therefore, we believed that GPR176 knockdown promoted pyroptosis and suppressed the EMT of breast cancer by increasing the levels of Zeb1, Slug, Snail, and Twist1. MMPs and VEGF are well known to break the extracellular matrix and promote metastasis [23]. GPR176 silencing was shown to reduce the expression of MMP1, MMP9, and VEGF, accounting for the effects of GPR176 in promoting the invasion and metastasis of breast cancer cells.

In summary, GPR176 is believed to be involved in the pathogenesis and subsequent progression of breast cancer by promoting the proliferation, anti-apoptosis, glucose catabolism, migration, and invasion of breast cancer cells. Its aberrant expression and methylation might be employed as biomarkers to indicate the aggressive behaviors and poor prognosis of breast cancer.

## Data Availability

Data sets used and/or analyzed during the current study are available from corresponding author upon reasonable request.
